# Stratification bias in low signal microarray studies

**DOI:** 10.1186/1471-2105-8-326

**Published:** 2007-09-02

**Authors:** Brian J Parker, Simon Günter, Justin Bedo

**Affiliations:** 1 Statistical Machine Learning Group, NICTA, Canberra, Australia; 2 Life Sciences Group, NICTA, Melbourne, Australia; 3 Research School of Information Sciences and Engineering, Australian National University, Canberra, Australia

## Abstract

**Background:**

When analysing microarray and other small sample size biological datasets, care is needed to avoid various biases. We analyse a form of bias, stratification bias, that can substantially affect analyses using sample-reuse validation techniques and lead to inaccurate results. This bias is due to imperfect stratification of samples in the training and test sets and the dependency between these stratification errors, i.e. the variations in class proportions in the training and test sets are negatively correlated.

**Results:**

We show that when estimating the performance of classifiers on low signal datasets (i.e. those which are difficult to classify), which are typical of many prognostic microarray studies, commonly used performance measures can suffer from a substantial negative bias. For error rate this bias is only severe in quite restricted situations, but can be much larger and more frequent when using ranking measures such as the receiver operating characteristic (ROC) curve and area under the ROC (AUC). Substantial biases are shown in simulations and on the van 't Veer breast cancer dataset. The classification error rate can have large negative biases for balanced datasets, whereas the AUC shows substantial pessimistic biases even for imbalanced datasets. In simulation studies using 10-fold cross-validation, AUC values of less than 0.3 can be observed on random datasets rather than the expected 0.5. Further experiments on the van 't Veer breast cancer dataset show these biases exist in practice.

**Conclusion:**

Stratification bias can substantially affect several performance measures. In computing the AUC, the strategy of pooling the test samples from the various folds of cross-validation can lead to large biases; computing it as the average of per-fold estimates avoids this bias and is thus the recommended approach. As a more general solution applicable to other performance measures, we show that stratified repeated holdout and a modified version of k-fold cross-validation, *balanced*, *stratified cross-validation and balanced leave-one-out cross-validation*, avoids the bias. Therefore for model selection and evaluation of microarray and other small biological datasets, these methods should be used and unstratified versions avoided. In particular, the commonly used (unbalanced) leave-one-out cross-validation should not be used to estimate AUC for small datasets.

## Background

When analysing microarray datasets for class comparison and class prediction [1] purposes, the generalisation performance of machine learning algorithms such as linear discriminant analysis (LDA) and support vector machines (SVM) is typically estimated using sample-reuse techniques, as the sample sizes are often too small to use a separate withheld test set: such schemes include k-fold cross-validation (CV), leave-one-out CV (LOOCV), bootstrap methods, or repeated holdout (also known as random splitting) [2-4]. The estimates of generalisation performance are then used for model selection, parameter tuning, feature selection, or performance evaluation in empirical comparisons of machine learning methods. When comparing the results of different classifiers it is also necessary to test for statistical significance [5,6]. Microarray data is atypical of the data commonly classified by machine learning methods as it often has a small sample size and low discriminability between the classes e.g. in cancer prognostic and therapeutic response studies. Various biases can occur in such a setting when using the above mentioned validation schemes [7]. For example, Ambroise and McLachlan [8] and Simon et al. [1] demonstrated an optimistic selection bias that occurs when gene selection is done using the entire dataset rather than separately for each resampled training set. This bias arises through incorporation of information from the test sets into the training of the classifier. Varma and Simon [9] demonstrated in a simulation study the optimistic hyperparameter selection bias [10] which occurs when reporting the best error rates achieved on the validation set used to tune classifier (hyper)parameters, rather than using a nested CV or separate test set to evaluate the classifier.

It is also known that in some limited situations, such as when measuring the error rate of a classifier on a perfectly balanced dataset, large pessimistic biases can occur when using CV [10,11] due to negatively correlated class proportions between the training sets and their corresponding test sets (where by pessimistic we mean that the measured performance is less than the true generalisation error). However, because of the limited circumstances in which this bias appears to be an issue (i.e. small, balanced, low signal datasets) it has been largely ignored in the literature.

What has not been previously appreciated is that this systematic bias due to inadequate stratification is quite pervasive and can occur in a wide variety of contexts, including when using ranking performance measures such as the frequently used area under the receiver operating characteristic curve (AUC). This is not obvious as the AUC is considered to be a measure that is insensitive to varying class proportions. Importantly, with these measures we show that the bias can also occur in highly imbalanced or skewed datasets, and indeed the effect of the stratification bias can be larger for these performance measures than for the standard error rate.

Many microarray studies have small datasets and weak signals (the signal strength here means the inherent discriminability of the signal, i.e. how well an optimal classifier can perform), thus the bias is especially important and steps should be taken to minimise it. We demonstrate several techniques including careful implementation of the AUC calculation and stratified variants of resampling schemes that can be used to remove or minimise the bias.

### Review of ROC and AUC calculation

For comparing and assessing the performance of gene selection and classification algorithms in the analysis of microarray and other biological datasets, the receiver operating characteristic curve (ROC) and the associated AUC are popular measures of performance [12]. They have several advantages over error rate [13-15] including insensitivity to the prior class probabilities and class-specific error costs. This is especially important in the case of microarray observational studies where the particular class proportions used may be unrelated to clinical prevalence, and in class comparison (i.e. differential gene expression) studies where an inherent measure of the discriminability of the signal using a given classifier is required. The ROC shows the trade-off between sensitivity and specificity for a two-class classifier or diagnostic system. It has long been used in medical diagnosis [16] and has become widely used in evaluating machine learning algorithms [17]. The AUC summarises an ROC and provides a single measure of the performance of a classifier and the discriminability of a signal: a random signal has an AUC of 0.5 and a perfectly discriminable signal has AUC of 1.0.

The AUC of a classifier with a scoring output, such as the probability of a sample being class 1, can be computed without first constructing an explicit ROC curve [18]:

AUC=S0−n+(n−+1)/2n+n−
 MathType@MTEF@5@5@+=feaafiart1ev1aaatCvAUfKttLearuWrP9MDH5MBPbIqV92AaeXatLxBI9gBaebbnrfifHhDYfgasaacH8akY=wiFfYdH8Gipec8Eeeu0xXdbba9frFj0=OqFfea0dXdd9vqai=hGuQ8kuc9pgc9s8qqaq=dirpe0xb9q8qiLsFr0=vr0=vr0dc8meaabaqaciaacaGaaeqabaqabeGadaaakeaacqWGbbqqcqWGvbqvcqWGdbWqcqGH9aqpdaWcaaqaaiabdofatnaaBaaaleaacqaIWaamaeqaaOGaeyOeI0IaemOBa42aaSbaaSqaaiabgUcaRaqabaGccqGGOaakcqWGUbGBdaWgaaWcbaGaeyOeI0cabeaakiabgUcaRiabigdaXiabcMcaPiabc+caViabikdaYaqaaiabd6gaUnaaBaaaleaacqGHRaWkaeqaaOGaemOBa42aaSbaaSqaaiabgkHiTaqabaaaaaaa@43AB@

where *n*_+ _and *n*_- _are the numbers of positive and negative samples, S0=∑iri
 MathType@MTEF@5@5@+=feaafiart1ev1aaatCvAUfKttLearuWrP9MDH5MBPbIqV92AaeXatLxBI9gBaebbnrfifHhDYfgasaacH8akY=wiFfYdH8Gipec8Eeeu0xXdbba9frFj0=OqFfea0dXdd9vqai=hGuQ8kuc9pgc9s8qqaq=dirpe0xb9q8qiLsFr0=vr0=vr0dc8meaabaqaciaacaGaaeqabaqabeGadaaakeaacqWGtbWudaWgaaWcbaGaeGimaadabeaakiabg2da9maaqahabaGaemOCai3aaSbaaSqaaiabdMgaPbqabaaabaGaemyAaKgabaaaniabggHiLdaaaa@368B@, and *r*_*i *_is the rank of the *i*th positive sample in the sorted classifier output (sorted in decreasing order of scores or posterior probabilities). Similarly, the ROC curve can be computed by an incremental algorithm which scans the sorted outputs [19]. The AUC is equivalent to the Wilcoxon-Mann-Whitney statistic, which measures the probability that two random samples from different classes will be ranked in the correct order [20]. An estimate of the standard error of the AUC is given analytically by the Hanley-McNeil estimate [20]. When combining the results of the folds or replicates of a validation procedure to compute an overall estimate of the ROC curves or AUC, there are two main approaches: the *pooling *and *averaging *strategies [10,13,19,21]. The pooling strategy involves collecting the classifier scoring outputs determined on each test set and calculating the AUC on this set of combined outputs. In the averaging strategy, a separate AUC is computed for each test set, and the mean of these AUCs is computed. Note that the pooling strategy is the only method of measuring AUC (and calculating ROC curves) when using LOOCV. Both strategies are used in practice and the current literature is equivocal about which approach is to be recommended (both approaches are described as valid estimates of AUC and ROC curves in [10,19,22]). Witten and Frank [10] note that the pooling strategy has the advantage that it is easier to implement. Also, it is expected to have a lower variance. In part, this is because when taking an average over *f *averages, each calculated over *n *results of a random variable *X*, we get the following variance: Varaverage(Xest)∝1f−1⋅n−1
 MathType@MTEF@5@5@+=feaafiart1ev1aaatCvAUfKttLearuWrP9MDH5MBPbIqV92AaeXatLxBI9gBaebbnrfifHhDYfgasaacH8akY=wiFfYdH8Gipec8Eeeu0xXdbba9frFj0=OqFfea0dXdd9vqai=hGuQ8kuc9pgc9s8qqaq=dirpe0xb9q8qiLsFr0=vr0=vr0dc8meaabaqaciaacaGaaeqabaqabeGadaaakeaacqWGwbGvcqWGHbqycqWGYbGCdaWgaaWcbaGaemyyaeMaemODayNaemyzauMaemOCaiNaemyyaeMaem4zaCMaemyzaugabeaakiabcIcaOiabdIfaynaaBaaaleaacqWGLbqzcqWGZbWCcqWG0baDaeqaaOGaeiykaKIaeyyhIu7aaSaaaeaacqaIXaqmaeaadaGcaaqaaiabdAgaMjabgkHiTiabigdaXaWcbeaakiabgwSixpaakaaabaGaemOBa4MaeyOeI0IaeGymaedaleqaaaaaaaa@4D16@. In contrast if we calculate a direct average over all results (like the pooling strategy) the variance will be lower: Varpool(Xest)∝1f⋅n−1
 MathType@MTEF@5@5@+=feaafiart1ev1aaatCvAUfKttLearuWrP9MDH5MBPbIqV92AaeXatLxBI9gBaebbnrfifHhDYfgasaacH8akY=wiFfYdH8Gipec8Eeeu0xXdbba9frFj0=OqFfea0dXdd9vqai=hGuQ8kuc9pgc9s8qqaq=dirpe0xb9q8qiLsFr0=vr0=vr0dc8meaabaqaciaacaGaaeqabaqabeGadaaakeaacqWGwbGvcqWGHbqycqWGYbGCdaWgaaWcbaGaemiCaaNaem4Ba8Maem4Ba8MaemiBaWgabeaakiabcIcaOiabdIfaynaaBaaaleaacqWGLbqzcqWGZbWCcqWG0baDaeqaaOGaeiykaKIaeyyhIu7aaSaaaeaacqaIXaqmaeaadaGcaaqaaiabdAgaMjabgwSixlabd6gaUjabgkHiTiabigdaXaWcbeaaaaaaaa@4737@. The difference in variance is only significant when *n *or *f *is small.

The pooling approach assumes that the results of each fold is a sample from the same population [21]. In the next section we show that this assumption is generally not valid for CV or bootstrap and can lead to large pessimistic biases.

## Methods

### Theoretical analysis

The fundamental issue is that most classifiers incorporate the prior probabilities of the classes, estimated from the training set, either as an explicit prior term, as is the case for generative classifiers such as LDA, or implicitly (see below). When using CV this estimate is negatively correlated with the class proportions of the corresponding test set. We now analyse the bias that arises from this negative correlation.

#### Analysis of bias in error rate and AUC estimation

CV uses sampling without replacement to partition the dataset into training and test sets, thus any deviation from the class proportions of the whole dataset in a training set leads to an opposite deviation in the corresponding test set. More specifically, the correlation of the class proportions of a training and test set pair is -1. For small datasets, unstratified CV can lead to large variations in the training and test set class proportions, so a large negative covariance between training and test sets class proportions exists. Stratified CV [10] is a CV variant which ensures that the proportions of the classes in the test sets of the folds are as close as possible to the overall class proportions. Stratified CV can reduce the above mentioned covariance but not remove it entirely due to irreducible quantisation errors which are significant for small test set sizes. For example, a 10-fold stratified CV on a dataset with 30 samples has three samples in each fold, and cannot be stratified correctly for two classes in equal proportions. Somewhat surprisingly, as shown in the empirical section of this paper, this tiny error can still lead to substantial biases. The same reasoning applies when using repeated holdout.

By contrast, bootstrap methods use sampling with replacement. A training set of the same size as the whole dataset is created by randomly sampling the whole dataset with replacement, with the remaining unsampled instances forming the test set. The test and training sets, excluding replicates, have a similar covariance of the class proportions as a 2 : 1 repeated holdout, although the added variance of the replicate samples leads to a somewhat smaller correlation of approximately -0.7.

Using Bayesian decision theoretic analysis, we show that this negative covariance between training and test set proportions can lead to large biases in error rate and AUC estimation. Let *f*_*k*_(*x*) be the class-conditional density of feature vector *x *in class *G *= *k*, and let *π*_*k *_be the prior probability of class *k*, such that ∑l=1Kπl=1
 MathType@MTEF@5@5@+=feaafiart1ev1aaatCvAUfKttLearuWrP9MDH5MBPbIqV92AaeXatLxBI9gBaebbnrfifHhDYfgasaacH8akY=wiFfYdH8Gipec8Eeeu0xXdbba9frFj0=OqFfea0dXdd9vqai=hGuQ8kuc9pgc9s8qqaq=dirpe0xb9q8qiLsFr0=vr0=vr0dc8meaabaqaciaacaGaaeqabaqabeGadaaakeaadaaeWaqaaGGaciab=b8aWnaaBaaaleaacqWGSbaBaeqaaaqaaiabdYgaSjabg2da9iabigdaXaqaaiabdUealbqdcqGHris5aOGaeyypa0JaeGymaedaaa@386A@, then Bayes theorem gives the posterior probabilities

Pr(G=k|X=x)=fk(x)πk∑l=1Kfl(x)πl
 MathType@MTEF@5@5@+=feaafiart1ev1aaatCvAUfKttLearuWrP9MDH5MBPbIqV92AaeXatLxBI9gBaebbnrfifHhDYfgasaacH8akY=wiFfYdH8Gipec8Eeeu0xXdbba9frFj0=OqFfea0dXdd9vqai=hGuQ8kuc9pgc9s8qqaq=dirpe0xb9q8qiLsFr0=vr0=vr0dc8meaabaqaciaacaGaaeqabaqabeGadaaakeaaieGacqWFqbaucqWFYbGCcqGGOaakcqWGhbWrcqGH9aqpcqWGRbWAcqGG8baFcqWGybawcqGH9aqpcqWG4baEcqGGPaqkcqGH9aqpdaWcaaqaaiabdAgaMnaaBaaaleaacqWGRbWAaeqaaOGaeiikaGIaemiEaGNaeiykaKccciGae4hWda3aaSbaaSqaaiabdUgaRbqabaaakeaadaaeWaqaaiabdAgaMnaaBaaaleaacqWGSbaBaeqaaOGaeiikaGIaemiEaGNaeiykaKIae4hWda3aaSbaaSqaaiabdYgaSbqabaaabaGaemiBaWMaeyypa0JaeGymaedabaGaem4saSeaniabggHiLdaaaaaa@53F6@

and, assuming the two class case with classes 1 and 2, the log ratio is

log⁡Pr⁡(G=1|X=x)Pr⁡(G=2|X=x)=log⁡f1(x)f2(x)+log⁡π1π2
 MathType@MTEF@5@5@+=feaafiart1ev1aaatCvAUfKttLearuWrP9MDH5MBPbIqV92AaeXatLxBI9gBaebbnrfifHhDYfgasaacH8akY=wiFfYdH8Gipec8Eeeu0xXdbba9frFj0=OqFfea0dXdd9vqai=hGuQ8kuc9pgc9s8qqaq=dirpe0xb9q8qiLsFr0=vr0=vr0dc8meaabaqaciaacaGaaeqabaqabeGadaaakeaacyGGSbaBcqGGVbWBcqGGNbWzdaWcaaqaaiGbccfaqjabckhaYjabcIcaOiabdEeahjabg2da9iabigdaXiabcYha8jabdIfayjabg2da9iabdIha4jabcMcaPaqaaiGbccfaqjabckhaYjabcIcaOiabdEeahjabg2da9iabikdaYiabcYha8jabdIfayjabg2da9iabdIha4jabcMcaPaaacqGH9aqpcyGGSbaBcqGGVbWBcqGGNbWzdaWcaaqaaiabdAgaMnaaBaaaleaacqaIXaqmaeqaaOGaeiikaGIaemiEaGNaeiykaKcabaGaemOzay2aaSbaaSqaaiabikdaYaqabaGccqGGOaakcqWG4baEcqGGPaqkaaGaey4kaSIagiiBaWMaei4Ba8Maei4zaC2aaSaaaeaaiiGacqWFapaCdaWgaaWcbaGaeGymaedabeaaaOqaaiab=b8aWnaaBaaaleaacqaIYaGmaeqaaaaaaaa@6548@

Note that as the signal becomes weaker, i.e. as the first term diminishes, the prior probabilities assume increased relative importance. The Bayes decision rule for the two class case is to classify a sample as class 1 when the likelihood ratio *f*_1_(*x*)/*f*_2_(*x*) exceeds a threshold *t*, where for the Bayes (minimum error) rate *t *is the inverse ratio of the prior proportions, *π*_2_/*π*_1_.

First we consider the bias in error rate. During CV or bootstrap, the prior proportions used to determine the classification threshold are estimated from the training set, π1train/π2train
 MathType@MTEF@5@5@+=feaafiart1ev1aaatCvAUfKttLearuWrP9MDH5MBPbIqV92AaeXatLxBI9gBaebbnrfifHhDYfgasaacH8akY=wiFfYdH8Gipec8Eeeu0xXdbba9frFj0=OqFfea0dXdd9vqai=hGuQ8kuc9pgc9s8qqaq=dirpe0xb9q8qiLsFr0=vr0=vr0dc8meaabaqaciaacaGaaeqabaqabeGadaaakeaaiiGacqWFapaCdaqhaaWcbaGaeGymaedabaGaeeiDaqNaeeOCaiNaeeyyaeMaeeyAaKMaeeOBa4gaaOGaei4la8Iae8hWda3aa0baaSqaaiabikdaYaqaaiabbsha0jabbkhaYjabbggaHjabbMgaPjabb6gaUbaaaaa@4112@, but due to incomplete stratification this will typically differ from the prior proportions of the whole data set, π1true/π2true
 MathType@MTEF@5@5@+=feaafiart1ev1aaatCvAUfKttLearuWrP9MDH5MBPbIqV92AaeXatLxBI9gBaebbnrfifHhDYfgasaacH8akY=wiFfYdH8Gipec8Eeeu0xXdbba9frFj0=OqFfea0dXdd9vqai=hGuQ8kuc9pgc9s8qqaq=dirpe0xb9q8qiLsFr0=vr0=vr0dc8meaabaqaciaacaGaaeqabaqabeGadaaakeaaiiGacqWFapaCdaqhaaWcbaGaeGymaedabaGaeeiDaqNaeeOCaiNaeeyDauNaeeyzaugaaOGaei4la8Iae8hWda3aa0baaSqaaiabikdaYaqaaiabbsha0jabbkhaYjabbwha1jabbwgaLbaaaaa@3E8C@. The corresponding test set proportions are negatively correlated, and so if π1train/π2train>π1true/π2true
 MathType@MTEF@5@5@+=feaafiart1ev1aaatCvAUfKttLearuWrP9MDH5MBPbIqV92AaeXatLxBI9gBaebbnrfifHhDYfgasaacH8akY=wiFfYdH8Gipec8Eeeu0xXdbba9frFj0=OqFfea0dXdd9vqai=hGuQ8kuc9pgc9s8qqaq=dirpe0xb9q8qiLsFr0=vr0=vr0dc8meaabaqaciaacaGaaeqabaqabeGadaaakeaaiiGacqWFapaCdaqhaaWcbaGaeGymaedabaGaeeiDaqNaeeOCaiNaeeyyaeMaeeyAaKMaeeOBa4gaaOGaei4la8Iae8hWda3aa0baaSqaaiabikdaYaqaaiabbsha0jabbkhaYjabbggaHjabbMgaPjabb6gaUbaakiabg6da+iab=b8aWnaaDaaaleaacqaIXaqmaeaacqqG0baDcqqGYbGCcqqG1bqDcqqGLbqzaaGccqGGVaWlcqWFapaCdaqhaaWcbaGaeGOmaidabaGaeeiDaqNaeeOCaiNaeeyDauNaeeyzaugaaaaa@53F8@, then π1test/π2test<π1true/π2true
 MathType@MTEF@5@5@+=feaafiart1ev1aaatCvAUfKttLearuWrP9MDH5MBPbIqV92AaeXatLxBI9gBaebbnrfifHhDYfgasaacH8akY=wiFfYdH8Gipec8Eeeu0xXdbba9frFj0=OqFfea0dXdd9vqai=hGuQ8kuc9pgc9s8qqaq=dirpe0xb9q8qiLsFr0=vr0=vr0dc8meaabaqaciaacaGaaeqabaqabeGadaaakeaaiiGacqWFapaCdaqhaaWcbaGaeGymaedabaGaeeiDaqNaeeyzauMaee4CamNaeeiDaqhaaOGaei4la8Iae8hWda3aa0baaSqaaiabikdaYaqaaiabbsha0jabbwgaLjabbohaZjabbsha0baakiab=Xda8iab=b8aWnaaDaaaleaacqaIXaqmaeaacqqG0baDcqqGYbGCcqqG1bqDcqqGLbqzaaGccqGGVaWlcqWFapaCdaqhaaWcbaGaeGOmaidabaGaeeiDaqNaeeOCaiNaeeyDauNaeeyzaugaaaaa@5167@. Hence, as noted by Kohavi [11], for a no-signal balanced dataset (i.e. equal class proportions), where the likelihood ratio and the ratio of prior proportions of the whole dataset, π1true/π2true
 MathType@MTEF@5@5@+=feaafiart1ev1aaatCvAUfKttLearuWrP9MDH5MBPbIqV92AaeXatLxBI9gBaebbnrfifHhDYfgasaacH8akY=wiFfYdH8Gipec8Eeeu0xXdbba9frFj0=OqFfea0dXdd9vqai=hGuQ8kuc9pgc9s8qqaq=dirpe0xb9q8qiLsFr0=vr0=vr0dc8meaabaqaciaacaGaaeqabaqabeGadaaakeaaiiGacqWFapaCdaqhaaWcbaGaeGymaedabaGaeeiDaqNaeeOCaiNaeeyDauNaeeyzaugaaOGaei4la8Iae8hWda3aa0baaSqaaiabikdaYaqaaiabbsha0jabbkhaYjabbwha1jabbwgaLbaaaaa@3E8C@, = 1, the opposite decision to the correct classification for the test set elements will be reached, leading to a pessimistic bias and worse-than-random results. That is, for a no-signal dataset the optimal Bayes classifier is a majority-voter, and in this case, the majority class on the training set is the opposite to that of the test set.

If there is a signal (i.e. the first term of eq. 1 > 0), then the classification accuracy will be pessimistically biased by this effect to a lesser extent, depending on the signal strength.

In the above analysis, the stratification bias for error rate only manifested when the dataset was balanced, however it can occur more widely in microarray studies. The aim in class comparison studies of microarrays is to determine the inherent discriminability of the genes in distinguishing the classes. A preprocessing technique in this case is to balance the classes by subsampling the majority class so that only the feature information is used in discriminating the classes and not the prior probabilities. Another technique applicable to classifiers that minimise regularised risk, such as Support Vector Machines (SVMs), is to use class dependent regularisation inversely proportional to the class proportions. Otherwise, for very imbalanced classes the classifier can effectively turn into a majority voter where no feature information is actually used to distinguish the classes. Note that with such preprocessing, the bias in error rate will also occur for imbalanced datasets.

Now we consider the bias in AUC estimation using the pooling strategy. An equivalent formulation of the Bayes decision rule described above for the two class case is that the posterior probability Pr(*G *= *k*|*X *= *x*) exceeds a threshold *t*, where for the Bayes decision rule *t *= 0.5. For a given trained classifier, varying the threshold in either of these formulations across its full range will generate equivalent ROC curves, and hence AUC. As the ROC curve measures the inherent discriminability of the class conditional distributions irrespective of the prior class probabilities, calculation using the likelihood ratio formulation would be preferred [22]. In practice, however, most classifiers will return an estimate of the posterior probability, or a related uncalibrated measure such as the decision value, or distance to the hyperplane separating the classes, in SVMs, and so this second formulation is typically used.

The pooling strategy for AUC calculation assumes that the classifier outputs across folds of CV are comparable and thus can be globally ordered. This is the case if the AUC is calculated using the likelihood ratio for ranking; if the AUC is calculated using posterior probabilities (or decision values) for ranking as is usually done, the prior probabilities will vary in each training set because of incomplete stratification, and hence the classifier outputs will not be comparable across folds. This can lead to test samples in different folds being ranked in the wrong order:

Assume a two-class classification problem with classes 1 and 2, using the posterior probabilities (of being class 1) for ranking the samples. Let π1train/π2train=δ⋅(π1true/π2true)
 MathType@MTEF@5@5@+=feaafiart1ev1aaatCvAUfKttLearuWrP9MDH5MBPbIqV92AaeXatLxBI9gBaebbnrfifHhDYfgasaacH8akY=wiFfYdH8Gipec8Eeeu0xXdbba9frFj0=OqFfea0dXdd9vqai=hGuQ8kuc9pgc9s8qqaq=dirpe0xb9q8qiLsFr0=vr0=vr0dc8meaabaqaciaacaGaaeqabaqabeGadaaakeaaiiGacqWFapaCdaqhaaWcbaGaeGymaedabaGaeeiDaqNaeeOCaiNaeeyyaeMaeeyAaKMaeeOBa4gaaOGaei4la8Iae8hWda3aa0baaSqaaiabikdaYaqaaiabbsha0jabbkhaYjabbggaHjabbMgaPjabb6gaUbaakiabg2da9iab=r7aKjabgwSixlabcIcaOiab=b8aWnaaDaaaleaacqaIXaqmaeaacqqG0baDcqqGYbGCcqqG1bqDcqqGLbqzaaGccqGGVaWlcqWFapaCdaqhaaWcbaGaeGOmaidabaGaeeiDaqNaeeOCaiNaeeyDauNaeeyzaugaaOGaeiykaKcaaa@599C@ where π1true/π2true
 MathType@MTEF@5@5@+=feaafiart1ev1aaatCvAUfKttLearuWrP9MDH5MBPbIqV92AaeXatLxBI9gBaebbnrfifHhDYfgasaacH8akY=wiFfYdH8Gipec8Eeeu0xXdbba9frFj0=OqFfea0dXdd9vqai=hGuQ8kuc9pgc9s8qqaq=dirpe0xb9q8qiLsFr0=vr0=vr0dc8meaabaqaciaacaGaaeqabaqabeGadaaakeaaiiGacqWFapaCdaqhaaWcbaGaeGymaedabaGaeeiDaqNaeeOCaiNaeeyDauNaeeyzaugaaOGaei4la8Iae8hWda3aa0baaSqaaiabikdaYaqaaiabbsha0jabbkhaYjabbwha1jabbwgaLbaaaaa@3E8C@ is the overall class proportions in the full dataset and *δ *may not equal 1 due to incomplete stratification. Consider any fold. Suppose *δ *> 1, i.e. class 1 is over-represented in the training set. Then the prior term of eq. 1 is log⁡π1train/π2train=log⁡π1true/π2true+log⁡δ
 MathType@MTEF@5@5@+=feaafiart1ev1aaatCvAUfKttLearuWrP9MDH5MBPbIqV92AaeXatLxBI9gBaebbnrfifHhDYfgasaacH8akY=wiFfYdH8Gipec8Eeeu0xXdbba9frFj0=OqFfea0dXdd9vqai=hGuQ8kuc9pgc9s8qqaq=dirpe0xb9q8qiLsFr0=vr0=vr0dc8meaabaqaciaacaGaaeqabaqabeGadaaakeaacyGGSbaBcqGGVbWBcqGGNbWziiGacqWFapaCdaqhaaWcbaGaeGymaedabaGaeeiDaqNaeeyyaeMaeeyAaKMaeeOBa4gaaOGaei4la8Iae8hWda3aa0baaSqaaiabikdaYaqaaiabbsha0jabbkhaYjabbggaHjabbMgaPjabb6gaUbaakiabg2da9iGbcYgaSjabc+gaVjabcEgaNjab=b8aWnaaDaaaleaacqaIXaqmaeaacqqG0baDcqqGYbGCcqqG1bqDcqqGLbqzaaGccqGGVaWlcqWFapaCdaqhaaWcbaGaeGOmaidabaGaeeiDaqNaeeOCaiNaeeyDauNaeeyzaugaaOGaey4kaSIagiiBaWMaei4Ba8Maei4zaCMae8hTdqgaaa@6171@, and log *δ *> 0. Thus, the samples in the test set of this fold will overall receive a higher ranking than in a completely stratified fold.

However, due to the negative correlation between the training and test set proportions, π1test/π2test=γ⋅(π1true/π2true)
 MathType@MTEF@5@5@+=feaafiart1ev1aaatCvAUfKttLearuWrP9MDH5MBPbIqV92AaeXatLxBI9gBaebbnrfifHhDYfgasaacH8akY=wiFfYdH8Gipec8Eeeu0xXdbba9frFj0=OqFfea0dXdd9vqai=hGuQ8kuc9pgc9s8qqaq=dirpe0xb9q8qiLsFr0=vr0=vr0dc8meaabaqaciaacaGaaeqabaqabeGadaaakeaaiiGacqWFapaCdaqhaaWcbaGaeGymaedabaGaeeiDaqNaeeyzauMaee4CamNaeeiDaqhaaOGaei4la8Iae8hWda3aa0baaSqaaiabikdaYaqaaiabbsha0jabbwgaLjabbohaZjabbsha0baakiabg2da9iab=n7aNjabgwSixlabcIcaOiab=b8aWnaaDaaaleaacqaIXaqmaeaacqqG0baDcqqGYbGCcqqG1bqDcqqGLbqzaaGccqGGVaWlcqWFapaCdaqhaaWcbaGaeGOmaidabaGaeeiDaqNaeeOCaiNaeeyDauNaeeyzaugaaOGaeiykaKcaaa@5718@, where *γ *< 1 i.e. class 1 is under-represented and class 2 is over-represented in the test set. This means that proportionally more samples of class 2 get this higher ranking than samples of class 1. By symmetry, the reverse applies when *δ *< 1, i.e. in the test sets where class 1 is over-represented, samples get lower rankings.

As the AUC is an estimate of the probability of two independent samples being ranked in the correct order, it can be seen that this will lead to a pessimistic bias of the AUC towards 0 if the samples are ranked together as in the pooling method. Note that this bias becomes more substantial as the predictions become less certain; when the predictor is a constant uncertain predictor (i.e. *P*(*G *= *i*|*X *= *x*) = 0.5, *i *∈ 1, 2) the bias is large and leads to an AUC less than the expected 0.5.

In contrast, the stratification bias discussed here does not affect AUC calculated using the averaging strategy which averages the AUC computed per test set over all folds. Within each test set, the AUC is insensitive to class proportions and so is accurate, and as a rank ordering between folds is not required, no bias will occur in the overall AUC estimate.

#### Application to practical induction algorithms

The classifier resulting from the Bayesian decision theoretic analysis, the Bayes classifier, is the optimal classifier. Practical induction algorithms aim to approximate the Bayesian decision theoretic analysis described above to varying extents, and suffer from stratification bias to varying degrees. A generative classifier such as LDA approximates this analysis by effectively estimating an underlying model incorporating class conditional likelihoods and prior class proportions as described above [23]. For LDA the class conditional likelihoods are modelled as multivariate Gaussians. Therefore, as analysed in the previous section, the degree of stratification bias depends on the signal strength: for low signal datasets, the relative importance of the prior term increases and so the relative stratification bias increases. In the limit of a no-signal dataset, only the prior term remains and the classifier becomes a simple majority voter with maximal stratification bias.

Some variants of LDA assume that the prior proportions are the same for all classes, and so the prior term is ignored. Such classifiers include minimum distance (aka nearest centroid) and nearest Mahalanobis distance classifiers [23], which are simple versions of LDA that classify a sample to the nearest class mean. As they exclude the prior term, they are not affected by the form of stratification bias described here. Other classifiers, including non-parametric classifiers such as k-nearest neighbour and discriminative classifiers such as SVMs, approximate the ideal Bayes classifier, including the prior term, indirectly. Such induction algorithms still implicitly incorporate prior probability information by inducing a classifier optimised for imbalanced datasets, and in the limit for random signals they will tend towards a majority voter. This means that the above analysis applies as well to these classifiers and they suffer from stratification bias accordingly, but without an explicit calculation of prior proportions they are more difficult to study analytically. SVMs are discriminative in that they find a separating hyperplane between the two classes and do not directly estimate the class-conditional likelihoods and prior proportions of the classes; they can output for ranking purposes the internal decision values used for classification (normally the distances to the hyperplane separating the classes). Therefore SVMs are affected by the class prior probabilities only implicitly and incompletely due to shifts in the hyperplane with varying class proportions, and so are only partially affected by the form of stratification bias discussed here. SVMs that generate a posterior probability estimate [24] approximate the Bayesian analysis above and thus are expected to be substantially affected by this stratification bias. Note that while we have limited the analysis to the two-class problem, the same arguments apply in the multiclass case, and to multiclass extensions of AUC. In addition to the stratification bias discussed above, there is a similar additional bias that can affect the pooling strategy of AUC estimation. Any non-systematic noise across the folds or replicates of the validation scheme, due to slightly different classifiers learnt from the training sets, will perturb the ranking values or probabilities and attenuate the AUC towards the random value of 0.5 (as opposed to the bias towards an AUC of 0 for the correlated noise in stratification bias).

Also, note that this analysis assumes homogeneous data in each of the labelled classes, as is the typical assumption in machine learning algorithms. If the classes in fact are heterogeneous with unidentified subclasses, then these internal subclasses may themselves not be fully stratified across folds (even when using stratified versions of CV), and so a similar analysis would apply to the subclasses in this case, although affecting the first, likelihood, term of eq. 1 rather than the second, prior probability, term as analysed here. Further analysis of this form of stratification bias in such heterogeneous datasets is outside the scope of this paper and is a focus of future research.

#### Ameliorating stratification bias

As described above, the bias is introduced by the negative covariance of the training and test set class proportions across the folds or replicates of the validation scheme. Therefore, approaches to remove this bias are focused on removing this covariance.

As noted previously, for estimating AUC, the averaging strategy does not suffer from this stratification bias. A more general approach, applicable also to other performance measures, is to use a sample-reuse method that does not introduce these correlated class proportions. Stratified repeated holdout samples independently from each of the classes in the proportion of the original dataset [25]. Stratified bootstrap sampling [4] similarly ensures that the training set has classes in the same proportions as the original set by sampling with replacement separately from each class. As the covariance and correlation between a constant (here the training set class proportions) and another random variable (the test set proportions) equals 0, these approaches completely remove this form of stratification bias.

For cross-validation, although stratified CV [10] removes the bulk of the covariance, a significant amount can remain, especially for imbalanced datasets and small test set sizes. When the sample size for each class is not a multiple of the number of folds the stratification is incomplete, causing the proportion of samples in the training sets to differ. As shown in the empirical section, this residual covariance can lead to substantial biases when the test sets are small.

As noted above, if the class proportions in the training sets are constant across all folds, then they are uncorrelated with the test set class proportions and no stratification bias is present. This suggests a modification to the standard CV to eliminate the stratification bias: ensure that the training set class proportions are representative of the overall data class proportions but remain constant across all folds. We describe a new validation method, balanced, stratified CV (BSCV), which does this. It sub-samples to exclude a small number of random samples from the training set in each fold. Given a stratified k-fold cross validation (or LOOCV) partition, let *T*_*cf *_be the count of class *c *in the training set of fold *f*, and define *M*_*c *_= *min*_*f*_*T*_*cf *_to be the minimum count of class *c *in the training sets across all folds; then, in each training set *T*_*cf *_- *M*_*c *_samples are randomly deleted for each class *c*. As all classes will have count *M*_*c *_in each training set, there will be no correlation between training and test set class proportions, removing the bias. Furthermore, as the number of elements of each class varies by only 1 among the training sets for stratified CV, the maximum number of removed elements from each training set is the number of classes: in the two class case at most two samples are removed. A potential drawback is the slightly smaller training sets, however, as at most one sample per class is removed, the degradation in performance due to learning curve effects is minimal and is dominated by the stratification bias. The pseudo-code of BSCV is given in Algorithm 1. To implement BSCV only minor modifications to stratified CV are needed. BSCV applied to LOOCV is denoted as balanced LOOCV, as LOOCV cannot be otherwise stratified. In the case of balanced LOOCV, only a single sample needs to be removed from each training set.

**Input**: training sets *T*_*i *_of the *m *folds provided by stratified CV, number of classes *n*

**Output**: training sets *T*_*i *_of BSCV

**for ***i *= 1 **to ***n ***do**

   **for ***j *= 1 **to ***m ***do**

      *C*_*ij *_= |{*x *∈ *T*_*j*_|*x *is of class *i*}|

   **end for**

end for

**for ***i *= 1 **to ***n ***do**

   *M*_*i *_= min_*j *_*C*_*ij*_

end for

**for ***i *= 1 **to ***n ***do**

   **for ***j *= 1 **to ***m ***do**

      remove (*C*_*ij *_- *M*_*i*_) random samples of class *i *from *T*_*j*_

   **end for**

end for

Algorithm 1: BSCV algorithm

### Empirical analysis

To evaluate empirically the effect of the stratification bias analysed in the previous sections, we performed experiments using Monte Carlo simulation and a real-world dataset. The sample-reuse validation schemes examined included stratified and unstratified CV, LOOCV, and bootstrap. All CVs were 10-fold with no repetitions where not indicated otherwise. The zero estimator *ε*0 bootstrap version was used in the bootstrap experiments as the aim was to investigate the relative performance differences due to stratification bias; the 0.632 and 0.632+ estimators bootstrap versions [26] correct for the learning curve effect of their smaller training set by incorporation of the resubstitution error, which could confound the bias we are investigating.

For SVM, the AUC can be computed by ranking on either the decision values or the estimated posterior probabilities. In the simulation experiments, estimated posterior probabilities were used, while for the real-world examples, decision values were used.

**Figure 1:**
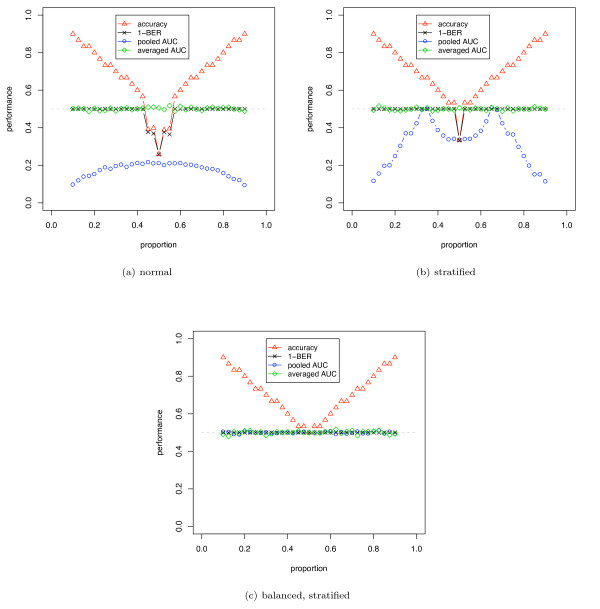
**Simulation results averaged over 500 runs using DLDA and versions of CV for a random signal**. The two classes have the same univariate Gaussian distribution (*d' *= 0), where the known mean and variance are used by the classifier. The number of samples is 30. The blue circles and green diamonds show the AUC computed using the pooling and averging strategy. The accuracy and balanced accuracy (1-BER) are shown as red triangles and black crosses.

**Figure 2:**
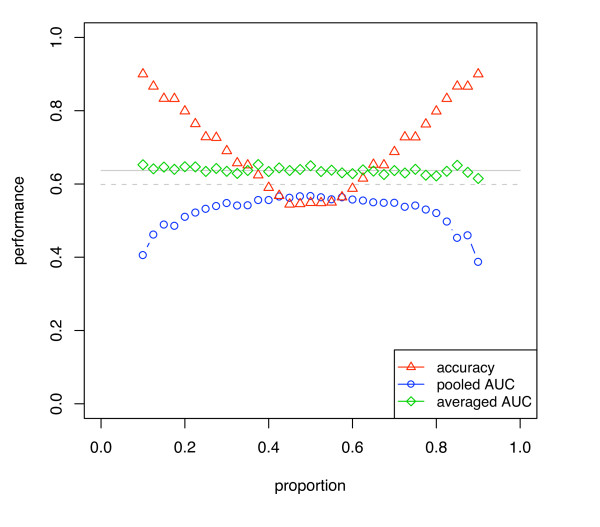
**Simulation results using DLDA and 10-fold unstratified CV for a weak signal**. Same experimental setup as in additional figure 1(a), but *d' *= 0.5.

**Figure 3:**
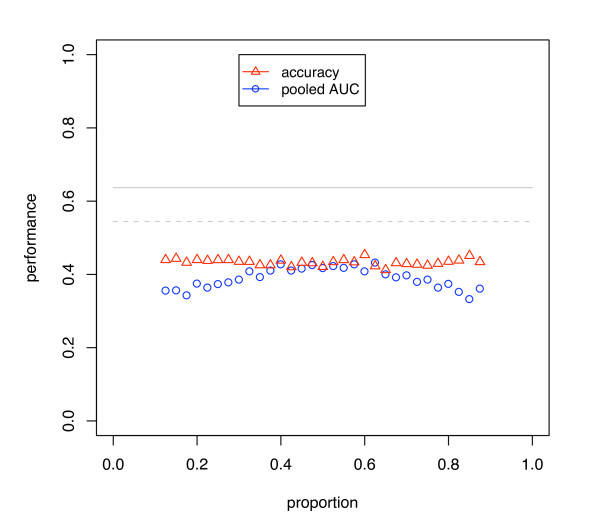
**Simulation results using weighted SVM**. The dataset was balanced through weighting by the inverse of the overall class proportions. Uses CV and SVM on a dataset of size 30 and discriminability *d' *= 0.5. The blue circles and red triangles show the AUC calculated using the pooling strategy and the classification accuracy.

**Figure 5:**
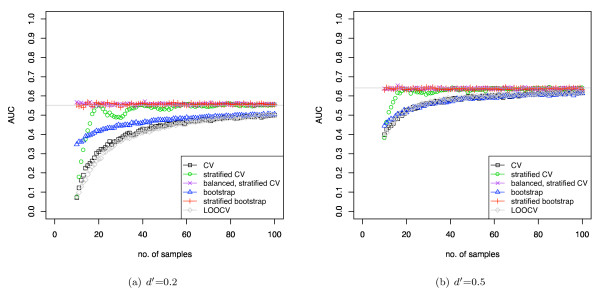
**Simulated classification results using DLDA (using known mean and variance) with varying separation of the means**. The two classes have a univariate Gaussian distribution.

**Figure 6:**
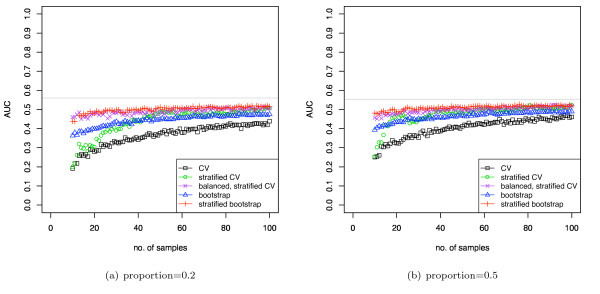
**Simulated classification results using DLDA for varying class proportions**. The two classes have a univariate Gaussian distribution and *d' *= 0.2. The mean and variance of the Gaussians were estimated from the data.

**Figure 7:**
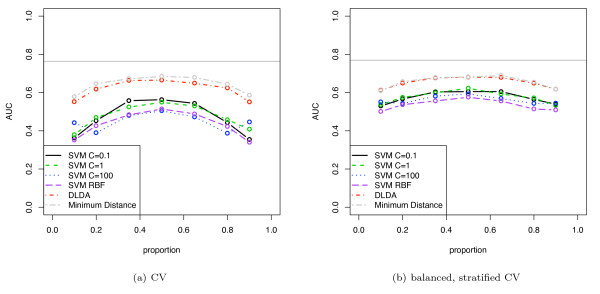
**Simulated classification results for various induction algorithms**. The two classes have a multivariate Gaussian distribution (10 dimensions). The discriminability *d' *is 1 and the data set contains 50 elements.

**Figure 8:**
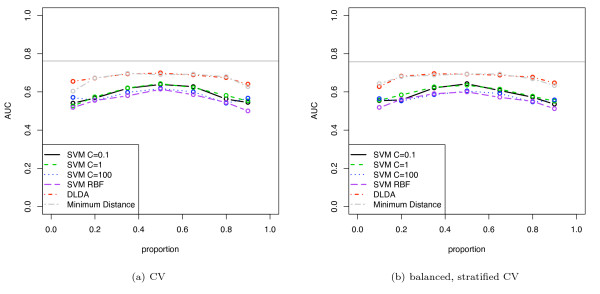
Same experimental setup as in figure 7, but AUC is calculated by averaging over the folds.

The grey lines in figures 1, 2, 3, 5, 6, 7 and 8 show the optimal values for AUC and the optimal (Bayes) error rate for class proportion 0.5. If both optimal AUC and error rate are shown, the line for AUC is solid. The R code and data for the experiments are available from the authors upon request.

#### Simulation

We performed Monte Carlo simulations using 1 and 10-dimensional normal distributions, the latter to approximately simulate ten significantly differentially expressed genes in a microarray study, as may be selected by some gene selection method (applied per fold). Samples were drawn from two multivariate Gaussian distributions (one Gaussian per class). The discriminability (or Mahalanobis distance) d′=(μ0−μ1)tΣ−1(μ0−μ1)
 MathType@MTEF@5@5@+=feaafiart1ev1aaatCvAUfKttLearuWrP9MDH5MBPbIqV92AaeXatLxBI9gBaebbnrfifHhDYfgasaacH8akY=wiFfYdH8Gipec8Eeeu0xXdbba9frFj0=OqFfea0dXdd9vqai=hGuQ8kuc9pgc9s8qqaq=dirpe0xb9q8qiLsFr0=vr0=vr0dc8meaabaqaciaacaGaaeqabaqabeGadaaakeaacuWGKbazgaqbaiabg2da9maakaaabaGaeiikaGccciGae8hVd02aaSbaaSqaaiabicdaWaqabaGccqGHsislcqWF8oqBdaWgaaWcbaGaeGymaedabeaakiabcMcaPmaaCaaaleqabaGaemiDaqhaaOGaeu4Odm1aaWbaaSqabeaacqGHsislcqaIXaqmaaGccqGGOaakcqWF8oqBdaWgaaWcbaGaeGimaadabeaakiabgkHiTiab=X7aTnaaBaaaleaacqaIXaqmaeqaaOGaeiykaKcaleqaaaaa@450C@ measures the degree of separation of the Gaussians. In our simulations we used *μ*_0 _= 0, Σ = *I *and varied *μ*_1 _to set the discriminability of the signal.

A *d' *of 0 is a random signal and corresponds to an error rate and AUC of 0.5; a *d' *of 0.5 is a weak signal; a *d'*of 1.0 is a moderate to strong signal; and a *d' *of 2.0 is a very strong signal. In the results section, we show the estimated error rate and optimal AUC corresponding to each simulation *d' *as a grey line. Two classes of induction algorithm were compared: diagonal LDA (DLDA) and SVMs. DLDA is a version of LDA that uses the assumption of independent features such that the covariance matrix is diagonal, which matches the simulation data model. Note that DLDA as defined here incorporates both the class conditional likelihood and prior proportion terms of eq. 1, as defined in [23], as opposed to the definition that excludes the prior term used in [2].

The performance measures examined were classification accuracy (1-error rate), AUC, and balanced error rate (BER). BER is an estimate of classifier performance independent of class proportions, defined as the mean of the false positive and false negative rates. In the simulations, we computed the optimal Bayes error rate and estimated the optimal AUC using Monte Carlo estimation from the known distributions. The simulations were repeated 500 times which was sufficient to generate small standard errors on the plots to allow the demonstration of the bias effects with statistical significance. Each bootstrap validation procedure used 10 replications.

#### Real-world dataset

We used the van 't Veer [27] breast cancer dataset as an example of a relatively weak-signal prognostic microarray study. The dataset contains 98 samples, with 52 in the poor prognosis (distant metastases developed in less than 5 years) class, and 46 in the good prognosis class. This dataset was classified using an SVM (linear kernel; C = 10) and all 5952 genes. The classifier output was the decision values and not posterior probabilities. The AUC was computed using both the pooling and averaging strategies. Random subsamples were used to generate curves of varying sample sizes.

A useful check for remaining bias in classifier evaluation is to perform a label permutation test. Permutation of the class labels yields a no-signal dataset with an expected random performance over multiple permutations. Any significant deviations of the mean performance from the expected random performance level are likely caused by some uncorrected bias; such a check will also detect the other forms of bias discussed in the introduction. The permutation test was applied for measuring AUC and error rate. In the case of error rate, the initial dataset was balanced by subsampling of the majority class to 46 samples, and stratified sampling of this balanced dataset was used, leading to an expected error rate of 0.5. Except where otherwise noted, at least 500 repetitions were performed for all experiments. Each bootstrap run consisted of 50 replicates.

## Results and discussion

### Simulation

Figure 1(a) shows an unstratified 10-fold CV estimate of AUC, accuracy and balanced accuracy (1-BER) for a size 30, simulated no-signal (*d' *= 0) univariate Gaussian dataset. The induction algorithm was DLDA, where the known distribution means and variances were used directly. The class proportions were varied from 0.1 to 0.9. For such a random dataset, the expected AUC and balanced accuracy is 0.5, and the expected accuracy at proportion 0.5 is 0.5 (and for data with majority class in proportion *p*, the expected accuracy is *p*). For proportions close to 0.5, the accuracy and balanced accuracy show a substantial pessimistic stratification bias below the expected 0.5 level. The AUC computed using the pooling strategy similarly shows a large pessimistic bias below the expected 0.5 and this occurs at all proportions, with a larger bias for imbalanced datasets. The AUC computed using the averaging strategy does not show any stratification bias, as expected, and lies on the 0.5 line. Stratified CV removes much of the stratification bias but there remains residual bias in accuracy, balanced accuracy and pooled AUC. Figure 1(c) shows the same situation as figure 1(a) but using balanced, stratified CV: BSCV shows no stratification bias.

Figure 2 shows a similar simulation to 1(a), but with a weak signal univariate Gaussian dataset (*d' *= 0.5). Note that the stratification biases for accuracy and AUC are still present but less than in figure 1. AUC computed using the averaging strategy shows no bias.

Figure 3 shows AUC and accuracy for a dataset corrected for class imbalance before analysis. A 5-dimensional *d' *= 0.5 signal was used with a linear SVM (*C *= 1) weighted to approximate a balanced dataset, by adjusting the weights parameter and the decision threshold by the inverse of the class proportions of the whole dataset; balancing the dataset by subsampling the majority class would give similar results. The results show that, in this situation, the stratification bias affecting the error rate occurs at all proportions indicating that it can occur more widely than may be expected.

**Figure 4:**
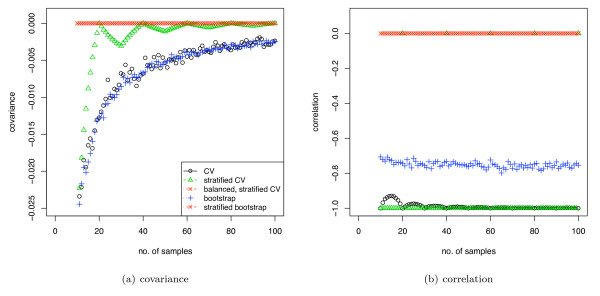
**Correlation and covariance of class proportions between the training and test sets**. The class proportions of the whole data sets are 0.5.

Figure 4 shows the covariance between the training and test set class proportions using various sample-reuse validation schemes. In general, the covariance decreases with increasing sample size. Unstratified cross-validation and unstratified bootstrap have the largest covariance, although the correlation of bootstrap is smaller due to the sampling with replacement. Also, note that with stratified CV, the covariance drops to zero when the sample size is a multiple of twice the fold number, in which case the data can be perfectly stratified, but for other sample sizes, some residual covariance remains. BSCV and stratified bootstrap, by contrast, remove the covariance completely.

Figure 5 shows AUC computed using the pooling strategy with DLDA and evaluated with various validation schemes. The classes of the simulated dataset have univariate Gaussian distributions with varying *d' *of 0.2 and 0.5 (*d' *of 1.0 and 2.0 are shown in supplementary figure 2 [see Additional file 1]), the sample sizes range from 10 to 100, and the class proportions are 0.5. Note that, as expected, the pessimistic bias for each validation method closely follows the covariance curves of figure 4(a). With increasing discriminability *d' *the bias decreases and the averaged performance measure becomes the same value for all validation schemes. The standard errors for the plots in figure 5 (over the 500 iterations) were lower than 0.01. The bias of CV was found to be statistically significant with *p *values lower than 0.001 up to a data set size of 60 (*d' *= 0.5).

Figure 6 shows the same data as figure 5(a), but for varying class proportions. Also, in this case the DLDA uses calculated estimates of mean and variance, and so is a more realistic simulation of DLDA classification. The BSCV obtained the highest AUC. Due to the parameter estimation task in this simulation, learning curve effects are now superimposed on the stratification bias, and so all curves show worse results at smaller sample sizes. Again the biases are highly statistically significant, as the standard errors of the plots were lower than 0.015 at all points. Note that the Hanley-McNeil estimate for the standard error of AUC on a single sample of the simulation 6(b) at sample size 50 is 0.08, and that the magnitude of the bias exceeds this, indicating that this bias could substantially affect performance evaluation of small, very low-signal datasets in practice.

Figure 7 shows the stratification bias for AUC computed for various induction algorithms: linear kernel SVM with cost parameter *C *varied from 0.1 to 100, SVM with radial basis function (*σ*^2 ^= 5), DLDA (with mean and variance estimated from the data), and a minimum distance classifier. The classes of the dataset had a 10-dimensional Gaussian distribution with *d' *= 1.0. The results for *d' *= 0.5 are available as supplementary figure 3 [see Additional file 1]. The dataset size was 50 and the pooling method was used to compute AUC.

The results for unstratified cross-validation (CV) shows that all classifiers are pessimistically biased compared with the BSCV results, indeed, many classifiers show AUCs below the random 0.5 level for imbalanced datasets. As expected, the minimum distance classifier, not incorporating prior proportions, does not show any significant stratification bias. Importantly, the relative ordering of the classifiers differs from CV to BSCV, e.g. using CV, linear SVM with *C *= 100 would appear to be worse than RBF SVM for not too extreme proportions while it is clearly better using BSCV. Thus, this experiment also demonstrates that model selection could be impacted by stratification bias.

Using BSCV, which removes the stratification bias, DLDA and minimum distance classifier have equal AUCs, as expected, and they are both the best performing classifiers (as their models match the simulation). The stratification bias of the SVMs is actually greater than that for DLDA in this simulation. This is due to the SVMs being less effective classifers on this dataset and showing a lower AUC. As discussed in the theoretical analysis section, with a weaker signal the prior proportions assume greater relative importance and thus leads to an increased stratification bias. For comparison, the Hanley-McNeil estimate of SE of the AUC for a single sample of the simulation at proportion 0.3 (and AUC = 0.6) is 0.085, and this is comparable to the bias shown in some SVMs at this class proportion. Note that all of the induction algorithms have somewhat lower AUC at the limits of the class proportions, even with BSCV: this is due in part to such imbalanced datasets being intrinsically more difficult to classify, and not only due to stratification bias. The standard errors of all plots were lower than 0.01. For all proportions and all classifiers, except the minimum distance classifier, the difference in AUC between CV and BSCV was found to be statistical significant with *p *values lower than 0.005.

We also reran the experiments using 10-times repeated CV and BSCV averaging results over 200 runs. The obtained graphs are almost identical to those in Figure 7 and are therefore not depicted. The standard deviations for the two representative classifiers when using non-repeated and repeated CV and BSCV are given in supplementary figure 1 [see Additional file 1]. The standard deviations decrease only moderately when using the 10-times repeated versions instead of single runs of CV and BSCV. The reason for this is that the noise between the different runs mainly stems from the difference in the data sets and not from the different separation of the data set into folds.

Figure 8 shows the same curves as figure 7 except that in this case AUC is computed using the averaging strategy. Note that all curves are substantially similar and unshifted across the two validation schemes, indicating that the averaging approach is unaffected by the form of stratification bias discussed here, as expected. The results for BSCV using the pooling method is substantially similar to the corresponding averaged AUC estimates, except that is slightly biased downwards due to inter-fold noise attenuating the pooling results.

### Real-world dataset

**Figure 9:**
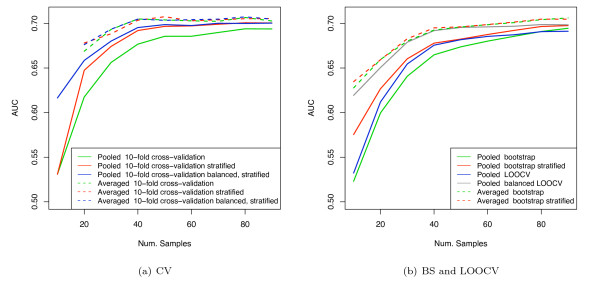
AUC estimates for van 't Veer breast cancer dataset using linear SVM.

**Figure 10:**
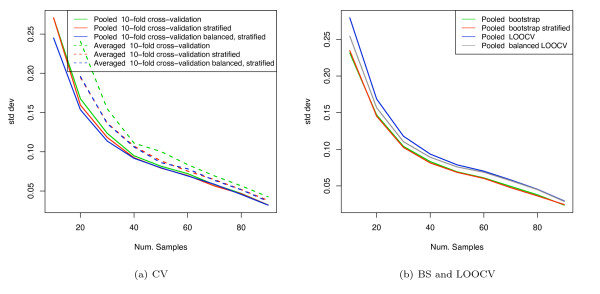
Standard deviations of AUC estimates for van 't Veer dataset using linear SVM.

Figure 9 shows the results of AUC estimation for the van 't Veer breast cancer dataset using SVM classification (with linear kernel), comparing both the pooling and averaging strategies. Figure 9(a) shows the results for cross-validation; 9(b) shows the results for LOOCV and bootstrap. The results for AUC calculation using a pooling strategy show that there is a substantial systematic pessimistic bias for the unstratified versions of cross-validation and bootstrap compared with the stratified versions. Also, stratified CV still shows some downward bias compared with balanced, stratified CV. LOOCV, which can only be used with a pooling strategy, shows substantial biases unless the balanced version, balanced LOOCV, is used. The performance of the balanced LOOCV is superior to the other pooled methods at small sample sizes as the training set of the folds is as large as possible. AUCs computed using the averaging strategy, by contrast, are not shifted relative to each other, when using either stratified or unstratified validation, and all reach the same asymptotic value, indicating that they do not suffer from the stratification bias. The results on the full dataset for the averaged AUC estimates are slightly higher than those for the pooling methods, including the stratified and balanced versions. This is due to the attenuation due to non-systematic classifier differences across folds, as described previously. Figure 10(a) and 10(b) show the standard deviations for figures 9(a) and 9(b), respectively. Note that the variance with stratified CV and balanced, stratified CV when using the averaging method is lower compared with unstratified CV, suggesting that such stratified validation schemes can give a worthwhile improvement in variance when used with averaged AUC estimation. Repeated cross validation would lower the variance further, as discussed in the simulation section. The Hanley-McNeil estimate for a sample size of 50 is 0.07, which approximately matches the empirical standard deviations at this sample size. The stratification bias at this sample size for linear SVM (C = 0.01) is substantially less than the SE in this case.

**Figure 11:**
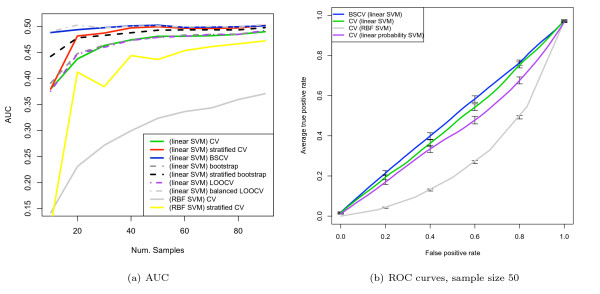
**AUC and ROC curve estimates for randomised van 't Veer dataset**. Error bars for ROC curves are 1 SE.

The next series of experiments used a randomised version of the van 't Veer dataset. Figure 11(a) shows the results for a linear kernel SVM using the pooling strategy for AUC estimation. Balanced, stratified CV and balanced LOOCV are approximately at the expected 0.5 line. Unstratified CV and LOOCV are pessimistically biased. Stratified CV shows some small remaining stratification bias for small sample sizes – figure 4 showed that stratified CV has some remaining covariance between training and test sizes and so this bias is expected. Note that stratified bootstrap also shows some remaining stratification bias for very small sample sizes. Although in theory stratified bootstrap has no covariance between training and test set sizes, the training set sizes are made constant by sample replication, and presumably the effect of a duplicate sample on the training of the classifier would be weaker than a truly independent pattern, and so the "effective" training set size of a class is less than the sample size would suggest (for example, consider 1-nearest neighbour: in 1-NN duplicates in the training set have absolutely no impact on the classification). Also, results for SVM with a radial basis function (RBF), aka Gaussian kernel, with parameter values C = 1 and *σ*^2 ^= 0.5 are presented. Only 100 random permutations were done with this SVM type due to the computational cost, which however is sufficient to demonstrate the bias. It is known that for such kernels, which increase the effective dimensionality of the data, the SVM will underfit and tend to a majority voter in large areas of the parameter space [28], and so rely maximally upon the prior proportion information; therefore it would be expected to perform poorly and suffer maximally from stratification bias. Indeed, figure 11(a) demonstrates very large pessimistic biases. The Hanley-McNeil SE estimate for sample size 50 is 0.08; note that the stratification bias for the RBF SVM exceeds the SE, indeed, some care would be needed to avoid confusing such a large negative AUC with a genuine signal. Note that stratified CV still shows substantial biases.

**Figure 12:**
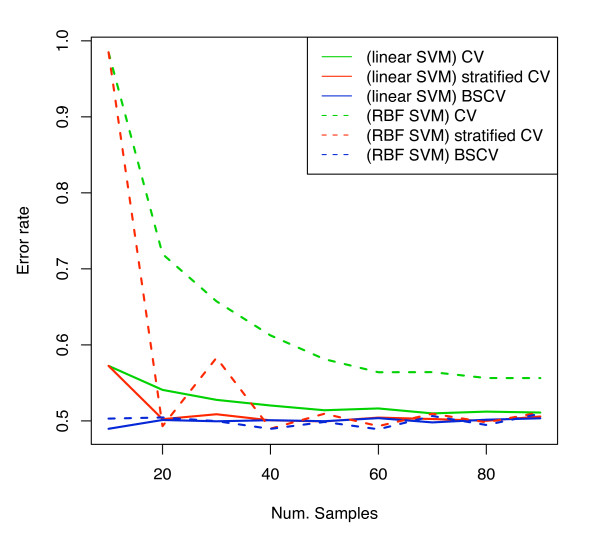
Error rate estimates for randomised van 't Veer dataset.

Figure 11(b) shows the ROC curves corresponding to figure 11(a) for 10-fold CV and a sample size of 50. The ROC curves were produced by pooling the samples of the folds of the cross-validation and computing a combined ROC curve. To generate confidence bounds, this was repeated 100 times and the vertically averaged curve displayed with standard error bars [29]. For CV the randomised dataset produces worse-than-random ROC curves with the pooling strategy, showing that the pooling strategy should also be avoided for ROC curve generation. With CV, the RBF kernel SVM shows large stratification biases, and the linear SVM using decision values shows only small biases at this sample size; BSCV applied to the linear SVM shows no substantial bias. Also shown are the results of the version of SVM returning posterior probabilities which, as expected, suffers more from stratification bias than the version using decision values. The final experiments investigated the effect of stratification bias on error rate. As above, a randomised version of the van 't Veer dataset was used. Figure 12 shows the results for both SVM kernels. BSCV is not biased and is at the expected 0.5 error rate; CV is the most pessimistically biased, and stratified CV removes most of the bias and is also at the expected 0.5 level, except for small sample sizes. The biases for the RBF kernel are especially substantial and larger than for the linear SVM. Note that stratified cross-validation still suffers from substantial bias in this case. Such large stratification biases of error rate could have an impact on model selection and evaluation, as the extent of the bias depends on the classifier type.

## Conclusion

In this paper we have analysed a previously under-appreciated bias which can strongly affect evaluation of small sample size (e.g. < 100), low-signal datasets typical of microarray studies. We showed that common sample-reuse validation schemes such as CV and bootstrap can lead to large pessimistic biases due to correlated class proportions between training and test sets.

We have performed a systematic study of this bias using simulation and a real-world dataset, and have demonstrated and evaluated how this bias can affect not only accuracy, but also AUC estimates. In low-signal microarray datasets, this bias can dominate the signal, and confound both model evaluation and model selection. In the case of model evaluation, while in practical applications a pessimistic bias can be, arguably, less detrimental than an optimistic bias, both are important to correct. A pessimistic bias could cause the rejection of a model which otherwise fulfills the required criteria. For example, when evaluating the effect of features in low-signal datasets in bioinformatics applications (e.g. whether a small gene set is predictive in microarray applications) a pessimistic bias can cause useful features to be misinterpreted or missed. In the case of model selection, the ranking of the performance of the models may change as different classifiers suffer from this stratification bias to differing extents, as noted in the experimental section (e.g. the minimum distance classifier is not affected by this bias and so may be favoured).

From the analysis in this paper, we have identified several approaches to ameliorate or remove this form of stratification bias and allow more accurate estimates of error rate and AUC for small, low-signal datasets: For AUC and ROC curve estimation, very substantial systematic stratification biases are introduced by the "pooling" estimation strategy; by contrast, we have shown that the "averaging" strategy of estimating the AUC per-fold avoids this bias and is the recommended approach. Note that (unbalanced) LOOCV, although commonly used for evaluating small datasets [7], can only use the pooling strategy and so is contraindicated when evaluating AUC. LOOCV can also exhibit a substantially biased error rate for weak signal, small, balanced, datasets and so should be avoided for such datasets.

As a general solution to remove this form of stratification bias, which can also be used with other performance measures including error rate and BER, we have demonstrated that the newly introduced balanced, stratified cross-validation and balanced LOOCV; stratified bootstrap; or stratified repeated holdout can avoid this stratification bias, and are recommended over their unstratified versions.

## Authors' contributions

BJP conceived the study. BJP, SG and JB drafted the manuscript and implemented the experiments. All authors read and approved the final manuscript.

## Supplementary Material

Additional file 1 Supplementary simulation results. Supplementary simulation results: Figure 1 shows standard deviations of single runs of two induction algorithms for standard BSCV and CV and 10-times repeated BSCV and CV; Figure 2 shows simulated classification results using DLDA with *d' *= 1.0 and *d' *= 2.0; Figure 3 shows simulated classification results for various induction algorithms for *d' *= 0.5. Click here for file
