# Supplementary material for: Stratification bias in low signal microarray studies
Source: BMC Bioinformatics. 2007 Sep 2;8:326. doi: 10.1186/1471-2105-8-326 (PMC2211509; doi:10.1186/1471-2105-8-326)
Supplement: Additional file 1 — Supplementary simulation results. Supplementary simulation results: Figure 1 shows standard deviations of single runs of two induction algorithms for standard BSCV and CV and 10-times repeated BSCV and CV; Figure 2 shows simulated classification results using DLDA with d' = 1.0 and d' = 2.0; Figure 3 shows simulated classification results for various induction algorithms for d' = 0.5. [file 1471-2105-8-326-S1.pdf]

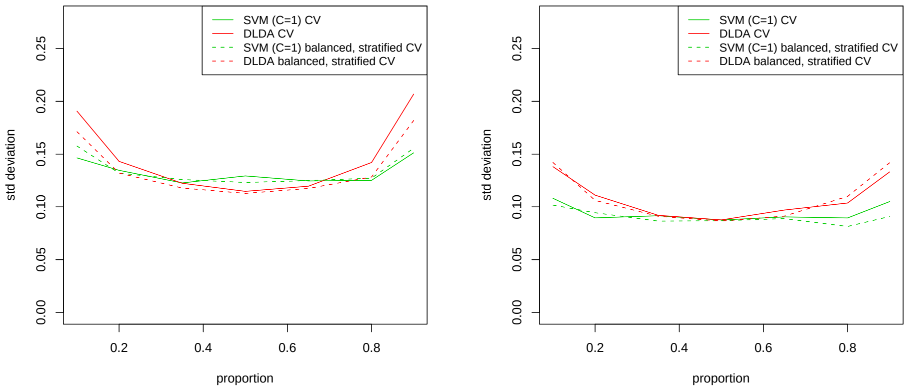

(a) standard CV, BSCV

(b) 10-times repeated CV, BSCV

Figure 1: Standard deviations of single runs of two induction algorithms for standard BSCV and CV and 10-times repeated BSCV and CV.

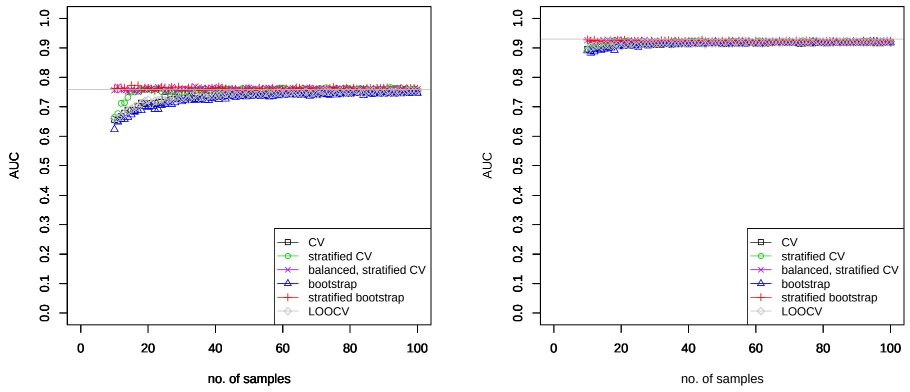

(a) d'=1

(b) d'=2

Figure 2: Simulated classification results using DLDA (using known mean and variance) with varying separation of the means.

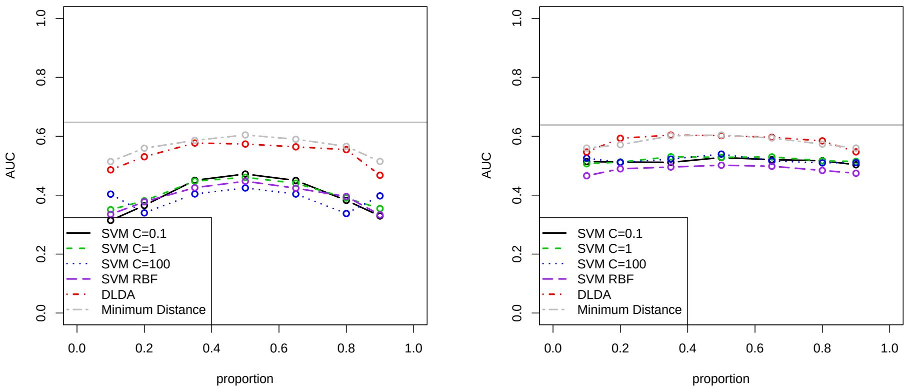

(a) CV (b) balanced, stratified CV

Figure 3: Simulated classification results for various induction algorithms. The two classes have a multivariate Gaussian distribution (10 dimensions). The discriminability d' is 0.5 and the data set contains 50 elements.
